# Retinol‐Loaded Mannosylerythritol Lipid Nanoliposome for Enhanced Human Skin Penetration

**DOI:** 10.1111/srt.70357

**Published:** 2026-06-05

**Authors:** Han‐woong Park, Dae‐Sung Yoo, Chan Jin Jeong, Soo Nam Park

**Affiliations:** ^1^ ASK Company Co. Ltd Daegu Republic of Korea; ^2^ Cosmetic Engineering Department of Biohealth Convergence College of Science and Convergence Technology Seoul Women's University Seoul Republic of Korea

**Keywords:** deformable liposome, mannosylerythritol lipid, retinol, skin barrier, transdermal delivery system

## Abstract

**Background:**

Functional cosmetic materials with anti‐aging properties, such as wrinkle reduction, often struggle to penetrate the skin's stratum corneum (skin barrier). Therefore, to exert anti‐aging effects, these materials must pass through the skin barrier and reach fibroblasts in the dermal layer. In this study, deformable liposomes were developed for efficient transdermal delivery of retinol, a well‐known anti‐wrinkle agent.

**Materials and Methods:**

Five formulations (ML‐1 to ML‐5) were prepared using hydrogenated lecithin (HL) and mannosylerythritol lipid (MEL) as an edge activator with HL:MEL mass ratios of 10:0, 7:3, 5:5, 3:7, and 0:10. Their physicochemical properties, including particle size, zeta potential, polydispersity, encapsulation efficiency and deformability index were analyzed. For the optimal formulation, the liposome morphology was observed using cryo‐TEM, and the retinol skin penetration ability of each liposome formulation was evaluated using a Franz diffusion cell.

**Results:**

Their physicochemical properties were investigated, and ML‐4 with an HL:MEL mass ratio of 3:7 was identified as the optimal formulation for transdermal delivery (particle size 89.3±6.8 nm, zeta potential −25.2±1.1 mV, polydispersity index 0.263±0.012, encapsulation efficiency 86.3±2.3%, and deformability index 5.76±1.9). Cryo‐TEM analysis confirmed the liposomal morphology of ML‐4. The retinol skin penetration ability of each formulation was evaluated using the Franz diffusion cell, with the conventional liposome (ML‐1) without MEL as the control. The ML‐4 formulation achieved a total retinol penetration of 29.0%, a 10.6% increase compared to ML‐1 (18.4%).

**Conclusions:**

In this study, the ML‐4 deformable liposome formulation, using MEL as an edge activator, efficiently encapsulates retinol, a wrinkle improvement agent, and improves skin penetration without causing physical damage. These findings suggest that ML‐4 can serve as an efficient transdermal delivery system for functional cosmetic materials.

**Abbreviations**
1,3‐BG: 1,3‐butylene glycol
CE: corneocyte envelope
Cer: ceramides
Chol: cholesterol
CLE: corneocyte lipid envelope
CMC: critical micelle concentration
CPE: corneocyte protein envelope
cryo‐TEM: cryogenic transmission electron microscopy
HL: hydrogenated lecithin
MEL: mannosylerythritol lipid
MLs: MEL‐liposomes
MMP: matrix metalloproteinase
PBS: phosphate‐buffered saline
PDI: polydispersity index
PGMD: polyglyceryl‐3‐methylglucose distearate
RA: Retinoic acid
ROS: reactive oxygen species
SDBS: sodium dodecyl benzene sulfonate
SDS: sodium dodecyl sulfate
TDDSs: transdermal delivery systems
UV‐vis: UV–visible

## 1 Introduction

The skin is composed of the epidermis, dermis, and subcutaneous fat layer, and the epidermis is divided into four layers: the basal layer, stratum spinosum, granular layer, and stratum corneum. In the epidermis, there are melanocytes that synthesize melanin in addition to keratinocytes. In the dermis, there are fibroblasts that express matrix metalloproteinase (MMP), which participates in the production or degradation of extracellular matrix components such as collagen. Keratinocytes undergo differentiation, during which their nuclei and organelles disappear and corneocytes with reduced water content accumulate to form the outermost layer of the skin the stratum corneum. The stratum corneum plays an important role as the skin's barrier. In particular, a series of biochemical processes in the maturation and desquamation of the stratum corneum are crucial for the skin's hydration and barrier function [1, 2, 3].

The stratum corneum has a “bricks and mortar” structure, which consists of corneocytes (bricks) and intercellular lipid lamellae between them [4]. Corneocytes are surrounded by the corneocyte envelope (CE), which consists of an inner protein envelope (CPE) and an outer lipid envelope (CLE). The lipid monolayer of the CLE is covalently attached to the CPE and functions as a support for the lamellar structure of the extracellular lipid matrix [3]. The main components of intercellular lipids, i.e., Cer, Chol, and fatty acids, exist at a molar ratio of approximately 1:1:1. Therefore, the amount and composition ratio of each component of intercellular lipids, as well as the CE composed of proteins and lipids, significantly affect the skin barrier function [5].

For a drug or active ingredient to be absorbed by the skin, it must first pass through the outermost layer of the epidermis—the stratum corneum. However, the stratum corneum functions as a barrier preventing the absorption of external substances, making penetration difficult [6]. There are three reported pathways through the stratum corneum: the intercellular lipid route through the lipid layers between the corneocytes, the intracellular route passing directly through the corneocytes, and the follicular route through the pores and sebaceous glands [6]. Therefore, it is considered necessary to develop transdermal delivery systems (TDDSs) such as liposomes using various physicochemical techniques and performing formulation studies to enhance the skin permeation of drugs and functional materials [7].

Liposomes are spherical vesicles composed of phospholipids. They consist of biocompatible components, can be decomposed in vivo, and have low toxicity. Liposomes are widely used in the manufacture of pharmaceuticals, cosmetics, and food products. Phospholipids, being amphiphilic, spontaneously form closed bilayer vesicles in water. This allows them to encapsulate both hydrophilic substances in the inner core and lipophilic substances within the bilayer membrane. However, liposomes have disadvantages such as physicochemical instability, a low skin absorption rate for normal skin, and lipid oxidation. To address these challenges and improve the permeation efficiency and stability of liposome formulations, it is essential to optimize various liposome characteristics, including composition, particle size, zeta potential, encapsulation efficiency, and formulation stability [8]. We previously performed a study on the stabilization of liposomes, in which liposomes self‐assembled with the polymers sodium hyaluronate and chitosan in a layer‐by‐layer manner were stabilized in proportion to the number of coating layers [9]. We also developed a capsosome that encapsulated a large number of liposomes within the coated polymer layers, enhancing the stability and applicability of the liposomes [10]. Additionally, studies on the characteristics and physicochemical stabilization of layer‐by‐layer self‐assembled liposomes using sodium alginate and chitosan were reported [11].

Liposomes are extensively utilized in cosmetic research to evalutate the dermal efficacy of functional bioactive agents. These materials exhibit potent antioxidant properties, facilitate anti‐aging (wrinkle reduction), inhibit hyperpigmentation (skin whitening), and reinforce skin barrier functions. Furthermore, various transdermal drug delivery systems (TDDSs) are being developed to enhance formulation stability and bioavailability. Our research group has previously reported diverse TDDS platforms, including transdermal absorption enhancers such as cell‐penetrating peptide liposomes [12], hydrogels [13], and liposome‐in‐hydrogel systems [14]. To improve the physicochemical stability of active ingredients, we have also investigated multilayer liposomes [9, 15], liposome‐core capsosomes [10], and solid lipid nanoparticles (SLNs) [16]. Additionally, stimuli‐responsive formulations, including pH‐sensitive hydrogels [17], pH‐sensitive liposomes [15], and reactive oxygen species (ROS)‐sensitive nanoparticles [18], have been engineered for targeted delivery.

In the early 1990s, conventional liposomes made from phosphatidylcholine were repeatedly found to be nearly incapable of overcoming the skin permeability barrier. Therefore, Cevc and Blume added a bilayer softening component known as an “edge activator,” specifically a biocompatible surfactant, to liposomes to increase the lipid flexibility and permeability of the vesicles and referred to these liposomes as “deformable liposomes” [19]. These liposomes are called elastic liposomes, deformable liposomes, or transfersomes [20, 21, 22].

In this study, mannosylerythritol lipid (MEL) was used as the edge activator for deformable liposomes. MEL—a biosurfactant produced by the microorganism Candida sp. SY16— has the full name 6‐O‐acetyl‐2,3‐di‐O‐alkanoyl‐β‐D‐mannopyranosyl‐(1→4)‐O‐meso‐erythritol [23]. MEL has two fatty acyl groups at the C‐2 and C‐3 positions of the mannose unit, an acetyl group at the C‐6 position, and erythritol at the C‐1 position. The hydrophobic part of MEL mainly consists of short‐chain hexanoic acid (49 mol%), dodecanoic acid (23 mol%), tetradecanoic acid (19 mol%), and the unsaturated fatty acid tetradecenoic acid (9 mol%) [23]. The critical micelle concentration (CMC) of MEL is 10 mg/L (1.5 × 10^−5^ M), which is lower than those of synthetic surfactants such as sodium dodecyl sulfate (SDS, 8.6 × 10^−3^ M) and sodium dodecyl benzene sulfonate (SDBS, 1.2 × 10^−3^ M) [24]. MEL has high biodegradability, low toxicity, good stability under various conditions, and excellent emulsifying activity [25, 26]. Additionally, it spontaneously self‐assembles into sponge (L3), bicontinuous cubic (V2), hexagonal (H2), and lamellar (Lα) nanostructures [27, 28]. It has been reported that MEL, owing to its ability to easily form lamellar (Lα) mesomorphic liquid crystals, exerts excellent moisturizing effects by retaining moisture at the intercellular level [29].

In the deformable liposome studied previously, synthetic surfactants such as polyethylene glycol (PEG) nonionic surfactants were used as edge activators. PEG‐based synthetic surfactants are rarely used in the cosmetics industry at present due to safety issues. Therefore, in this study, we used MEL biosurfactant, which has excellent physicochemical properties and efficacy as an edge activator as well as high compatibility with cosmetics applications, to manufacture deformable liposome and develop a new formulation that delivers anti‐aging agents deep into the skin.

The active ingredient used in the present study is retinol. Retinoids—a group of vitamin A derivatives—are among the most extensively researched ingredients in skincare for preventing aging and enhancing the appearance of mature skin. In South Korea, retinol is used as an anti‐wrinkle agent in functional cosmetics [30, 31]. It can stimulate collagen synthesis, inhibit MMP activity, reduce oxidative stress, and regulate gene expression [30, 32, 33]. Retinol has also shown efficacy in improving the visual signs of intrinsic and extrinsic aging, such as wrinkles and irregular pigmentation. Retinoic acid (RA) is the active form of vitamin A, and retinol acts as a precursor that is converted into RA, an active metabolite within the human skin. When retinol is applied topically to human skin, it penetrates the skin and is sequentially converted into retinaldehyde and then RA. Topical application of retinol not only increases the expression of type I collagen but also has remarkable anti‐aging effects on the skin, suggesting that retinol is a promising and safe natural anti‐aging material. Meanwhile, RA primarily functions through its effects on gene expression and cell differentiation, and it also influences melanin production and distribution [34, 35, 36]. RA is also known to improve hyperpigmentation by regulating melanin production, promoting cell differentiation, and suppressing inflammation [37].

**SCHEME 1:**
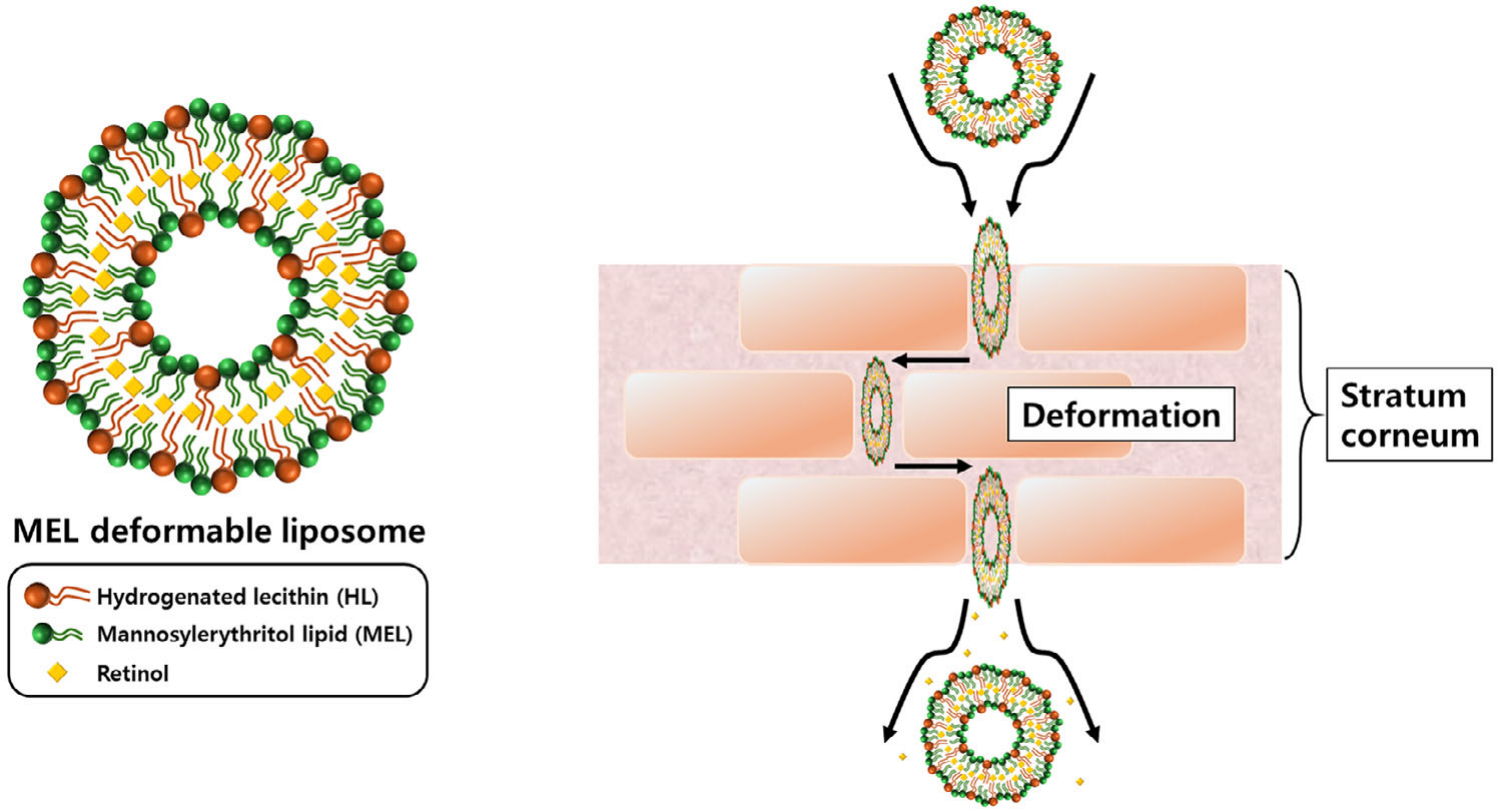
Skin permeation of MEL deformable liposome.

In this study, the MEL biosurfactant was used as an edge activator to produce deformable liposomes (or elastic liposomes) that encapsulate retinol, which is a functional substance for skin wrinkle improvement, as illustrated in Scheme 1. The physicochemical properties of the manufactured deformable liposomes, such as the zeta potential, storage stability, encapsulation efficiency, and deformability index, were verified using particle size and zeta potential analysis, UV‐vis spectrophotometry, mini‐extrusion, and cryogenic transmission electron microscopy (cryo‐TEM). We also used a Franz cell diffusion system to examine the depth, speed, and amount of skin penetration by the retinol in the deformable liposome and how quickly it was absorbed. The results indicated that the optimal MEL‐liposome formulation selected in this study is highly effective transdermal drug delivery system (TDDS), with potential applicability for delivering cosmetic functional materials, such as retinol, in cosmetics.

## 2 Materials and Methods

### 2.1 Materials

MEL was obtained from LABIO Co., Ltd. (Seoul, Korea), and hydrogenated lecithin (HL) was purchased from Lipoid GmbH (Germany). Cer was obtained from Doosan Co., Ltd. (Seoul, Korea). Cholesterol was obtained from Active Concepts (Lincolnton, North Carolina, USA). Retinol was obtained from BASF (Ludwigshafen, Germany). Chloroform and methanol of analytical grade were purchased from JT Baker (Phillipsburg, New Jersey, USA).

### 2.2 Preparation of MLs

MEL‐liposomes (MLs) were prepared via a thin‐film hydration method. The various formulations consisting of HL, MEL, Cer, Chol, and retinol are presented in Table 1.

**TABLE 1:** Compositions of the MLs.

Formulation code (HL:MEL mass ratio)		Composition (%, w/v)				
		HL	MEL	Cer	Chol	Retinol
**ML‐1**	(10:0)	0.50	—	0.005	0.1	0.05
**ML‐2**	(7:3)	0.35	0.15	0.005	0.1	0.05
**ML‐3**	(5:5)	0.25	0.25	0.005	0.1	0.05
**ML‐4**	(3:7)	0.15	0.35	0.005	0.1	0.05
**ML‐5**	(0:10)	—	0.50	0.005	0.1	0.05

Five MEL‐liposome formulations (ML‐1—ML‐5) were prepared with varying weight ratios of HL, a phospholipid, and MEL (used as an edge activator), as shown in Table 1. As listed in Table 1, all ingredients were dissolved in a chloroform/methanol mixture (5/5 v/v, 60 mL), and then, the solvents were removed using a rotary evaporator (IKA, Germany) to form a lipid film. All preparation procedures involving retinol were performed under light‐protected conditions using aluminum foil shielding, and sample containers were purged with nitrogen gas and processed rapidly to minimize oxidative degradation. This lipid film was hydrated with distilled water in an ultrasonic bath (Kudos, China) for 20 min to prepare a lipid suspension. This lipid suspension was treated with a microfluidizer (Micronox, Korea) and filtered through a 0.45 µm filter (Minisart CA 26 mm) to obtain the final liposome solution, which was used in the experiment.

### 2.3 Analysis of Particle Size and Zeta Potential

The particle‐size distribution and zeta potential of the MLs were measured using a particle‐size analyzer (Litesizer DLS, Anton Paar, Austria) and a zeta‐potential analyzer (Zetasizer Nano ZS, Malvern Instruments, United Kingdom), respectively. Average particle size was calculated using cumulative analysis and particle distribution was analyzed using the CONTIN algorithm [7]. The MLs were analyzed at 25°C, and all measurements were performed in triplicate.

### 2.4 Encapsulation Efficiency of MLs

The liposomes (1 mL, 10 mg mL^−1^) were purified via 0.45‐µm filtration to remove unencapsulated retinols. Subsequently, an excess amount of ethanol was added to dissolve the retinol contained in the filtered liposomes. After the ethanol was completely removed by a rotary evaporator, the sample was redissolved in ethanol (1 mL). All analytical procedures were conducted under light‐protected conditions using aluminum foil shielding, and sample containers were purged with nitrogen gas prior to analysis to prevent oxidative degradation of retinol. The retinol content in the MLs was measured at its maximum absorption wavelength of 325 nm using a UV‐visible (UV‐vis) spectrometer, and a calibration curve (µg/mL vs. absorbance) for retinol concentration was created using a standard solution. It was also confirmed by Beer's law, A = εbc (where A = absorbance, ε = molar extinction coefficient, and b = light penetration length; cm). To evaluate potential interference from formulation components, blank ML formulations without retinol were analyzed under identical conditions and showed negligible absorbance at 325 nm. Background correction was applied accordingly. Recovery was assessed by comparing the measured retinol content with the initial amount of retinol added during formulation. The concentration of the loaded retinol was then calculated, and the calculated value was substituted into the following Equation (1) to determine the entrapment efficiency.

Entrapment efficiency (%) = C_e_/C_i_ x 100 (1)

Here, *C_i_* represents the initial concentration of retinol (mg/mL), and *C_e_* represents the concentration of loaded retinol (mg/mL).

### 2.5 Evaluation of Deformability Index of MLs

To evaluate the deformability index of the prepared MLs, the degree of deformation when passing through an artificial permeation barrier was measured using a mini extruder (Avanti Polar Lipids). MLs were extruded through a polycarbonate membrane with a pore size of 50 nm at a pressure of 0.2 MPa for 1 min, and the amount of ML solution passing through the membrane was recorded. Additionally, the size of ML particles that passed through the membrane was measured. The deformability index of the ML membrane was determined using Equation (2) [38].

Deformability index = J_flux_ x (r_v_/r_p_)^2^ (2)

 where *J_Flux_* (mL/cm^2^/min) is the rate of nanoliposome mass transfer across the membrane per unit area over time, *r_v_* is the particle size of the nanoliposome after extrusion (nm), and *r_p_* is the pore size of the membrane (nm).

### 2.6 Cryo‐TEM

A cryogenic transmission electron microscope (cryo‐TEM; Cryo Tecnai F20, FEI Company, USA, 200 kV) was used to observe the morphology of the prepared deformable liposome. A drop of sample (no more than 3 µL) was placed on a grid (Lacey formvar/carbon, 200–300 mesh copper, Ted Pella, Redding, USA) and the sample was flash frozen in liquid ethane cooled with liquid nitrogen to prevent ice crystal formation using a Vitrobot (FEI Company, USA) apparatus and then stored in liquid nitrogen. Processed cryogenic transfer specimen holders (Model 626 DH, Gatan, USA) were first cooled to below 40°C using a baking cycle (Zeolite) with a dry pumping station (Model 655, Gatan, USA) for at least 2 h and then further cooled with liquid nitrogen (maintained at −178°C to −185°C). Sample grids stored in liquid nitrogen were mounted on cooled specimen holders and observed by cryo‐TEM [15]. The liposome bilayer thickness was calculated using the scale bar in the acquired cryo‐TEM image.

### 2.7 In Vitro Skin Permeation Using Franz Diffusion Cells

In vitro skin permeation studies of retinol‐loaded deformable liposomes were conducted using a Franz diffusion cell (DHC‐6TD, Logan Instruments, USA). Human cadaver skin (epidermis and dermis) obtained from the trunk region was supplied by a commercial vendor (Seedgroup Derma:Lab, Seoul, Republic of Korea). According to the supplier, the total skin thickness was approximately 700–800 µm, and the epidermal thickness was approximately 100 µm. Skin integrity testing was not performed by the supplier; therefore, integrity assessment was conducted at the experimental site, and skin samples showing visible damage were excluded prior to the experiment.

The skin samples were stored frozen at −20°C to −60°C and thawed at room temperature for approximately 10 min prior to use. Subcutaneous fat and excess connective tissue were carefully removed before mounting. The skin was mounted between the donor and receptor chambers with the stratum corneum facing the donor compartment and the dermal side facing the receptor compartment.

The effective diffusion area was approximately 1.77 cm^2^, corresponding to the 15 mm diameter Franz diffusion cell orifice. The receptor chamber was filled with 12 mL of HCO‐60:ethanol:phosphate‐buffered saline (PBS) (2:20:78, w/w) and maintained at 37°C under continuous stirring at 600 rpm. The donor chamber was loaded with 400 µL of the respective formulation. As a control, a retinol solution dissolved in 1,3‐butylene glycol (1,3‐BG) at the same concentration was used.

Receptor fluid samples were collected at predetermined time points. After 24 h, the skin surface was washed with PBS, and the stratum corneum was removed by tape stripping (three strips, Scotch tape, 3 M, USA). The collected tapes and remaining skin were extracted with 100% ethanol by sonication, and retinol content was quantified using a UV–vis spectrophotometer [1, 14].

### 2.8 Statistical Processing

All experiments were conducted in triplicate, and the results are expressed as mean ± standard deviation (SD). Statistical analyses were performed using GraphPad Prism 7.0 software (San Diego, CA, USA). Differences between the control group and the MEL‐liposome group were evaluated by one‐way analysis of variance (ANOVA). Differences were considered statistically significant at *p* < 0.05 (*n* = 3).

## 3 Results and Discussion

### 3.1 Particle Size, PDI, and Zeta Potential of MLs

MLs were prepared using HL and MEL at mass ratios of 10:0, 7:3, 5:5, 3:7, and 0:10, and these formulations were designated as ML‐1, ML‐2, ML‐3, ML‐4, and ML‐5, respectively (Table 1). The measurement results for the particle size, polydispersity index (PDI), and zeta potential of the MLs are presented in Table 2.

**TABLE 2:** Formulation of liposomes with different average particle sizes, PDIs, and zeta potentials.

Formulation code (HL:MEL mass ratio)		Size (nm)	PDI	Zeta potential (mV)
**ML‐1**	(10:0)	137.0 ± 34.4^a^	0.249 ± 0.034^a^	−32.9 ± 6.6^a^
**ML‐2**	(7:3)	79.5 ± 14.3^b^	0.268 ± 0.006^a^	−27.6 ± 1.9^ab^
**ML‐3**	(5:5)	87.5 ± 10.9^b^	0.271 ± 0.008^a^	−26.7 ± 2.3^ab^
**ML‐4**	(3:7)	89.3 ± 6.8^b^	0.263 ± 0.012^a^	−25.2 ± 1.1^b^
**ML‐5**	(0:10)	181.2 ± 54.3^a^	0.226 ± 0.055^a^	−8.1 ± 0.5^c^

Different letters (a, b and c) indicate significant differences (*p* < 0.05).

The average particle sizes of ML‐1, ML‐2, ML‐3, ML‐4, and ML‐5 were measured as 137.0 ± 34.4, 79.5 ± 14.4, 87.5 ± 10.9, 89.3 ± 6.8, and 181.2 ± 54.3 nm, respectively. The deformable liposomes (ML‐2, ML‐3, and ML‐4) had smaller particle sizes than the normal liposome (ML‐1) and MEL liposome (ML‐5), and the average particle size was <100 nm.

The PDI of liposomes determines their size homogeneity. PDI values of <0.3 and >0.3 correspond to monodisperse and polydisperse liposomes, respectively. Therefore, if the PDI of liposomes is <0.3, they have good size homogeneity [39]. The PDI values of ML‐1, ML‐2, ML‐3, ML‐4, and ML‐5 were 0.249 ± 0.0304, 0.268 ± 0.006, 0.271 ± 0.008, 0.263 ± 0.012, and 0.226 ± 0.055, respectively. As the PDI was <0.3 for all the ML formulations, the particle distribution exhibited monodispersity, indicating that the liposome particle size was homogeneous.

Using MEL as an edge activator, the particle size of the deformable liposome was small and stable even in a wide range of HL:MEL weight ratios (7:3 to 3:7). This suggests that MEL‐liposome may have greater applicability as a skin delivery system in cosmetics when compared to the HL:PGMD weight ratio (9:1 to 8:2) of deformable liposomes using polyglyceryl‐3‐methylglucose distearate (PGMD) as an edge activator in a previous study [14].

The liposomes’ zeta potential represents the overall charge that they acquire in a specific medium and is defined as the potential at the hydrodynamic shear boundary. A higher zeta potential predicts a more stable dispersion, implying that the particles in the suspension tend to repel each other, preventing aggregation. Generally, liposome suspensions with a zeta potential of +30 mV or higher or –30 mV or lower are considered stable. Furthermore, the zeta potential is related to the charge on the vesicle surface, which affects not only the stability of the liposomes but also their properties and their interaction with the skin. All the formulations prepared in this study (*n* = 3) exhibited negative zeta potentials: –32.9 ± 6.6 mV for ML‐1 (the normal liposome), –27.6 ± 1.9 mV for ML‐2, –26.7 ± 2.3 mV for ML‐3, –25.2 ± 1.1 mV for ML‐4, and –8.1 ± 0.5 mV for ML‐5 (MEL alone). As expected, owing to the ionic nature of the HL hydrophilic head, the normal liposome (ML‐1) composed solely of lecithin exhibited the most negative zeta potential among the liposome formulations, while the zeta potentials of ML‐2, ML‐3, and ML‐4, with decreasing lecithin ratios, exhibited a decreasing trend. ML‐5, corresponding to standalone MEL with a nonionic hydrophilic group and without lecithin having an ionic hydrophilic group, exhibited a low zeta potential, as expected.

The zeta potentials of ML‐2, ML‐3, and ML‐4 (excluding ML‐5) decreased slightly but exhibited similar values within the margin of error. This result suggests that the hydrophilic head of HL was primarily located on the outer layer of the double‐membrane of the ML. When liposomes are prepared using phospholipids through the thin‐film hydration method, phospholipids are first concentrated under reduced pressure to form a lipid film and then hydrated to form liposomes through self‐assembly. In the case of ML, it is assumed that the hydrophilic part of MEL, which is shorter than HL, is arranged closer to the liposome membrane than the hydrophilic group of HL, contributing to liposome formation. Therefore, it is considered that the hydrophilic part of HL is located further outside the liposome bilayer, affecting the Zeta potential value. For this reason, the zeta‐potential values of ML‐2, ML‐3, and ML‐4 exhibited little difference, but a decreasing trend was observed with a reduction in the HL content (and an increase in the MEL content).

### 3.2 Stabilization of MLs

**FIGURE 1:**
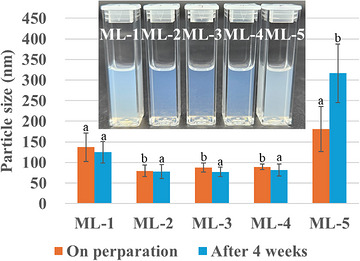
Stability of the particle size of the MLs after 4 weeks. The inset shows the physical appearance of the samples upon preparation; different letters (a and b) indicate significant differences (*p* < 0.05).

The stability of the MLs was assessed by comparing the particle sizes immediately after manufacture and after 4 weeks of storage (Figure 1). The measured changes in the average particle size after four weeks were as follows: ML‐1, reduction from 137.0 to 124.7 nm; ML‐2, reduction from 79.5 to 77.6 nm; ML‐3, reduction from 87.5 to 77.3 nm; ML‐4, reduction from 89.3 to 81.7 nm; ML‐5, increase from 181.2 to 316.7 nm. ML‐1, ML‐2, ML‐3, and ML‐4 exhibited slight reductions in average particle size after 4 weeks but maintained particle sizes similar to those immediately after manufacture, indicating relatively high stability. However, ML‐5 was discovered to be an unstable formulation, with the average particle size increasing by a factor of approximately 1.5 over 4 weeks.

The stability of particle size over a 4‐week storage period can be anticipated from the zeta‐potential results presented in Table 2. Generally, zeta potential values of −30 mV or lower (absolute value of ≥30 mV) indicate stability [23]. Except for ML‐5, all the MLs had a relatively stable absolute zeta potential in the range of –25 to –30 mV. ML‐5 had a low absolute zeta potential of –8 mV. This indicates that the repulsive force between the particles is weak, leading to particle aggregation and instability.

### 3.3 Encapsulation Efficiency of Retinol in ML

**FIGURE 2:**
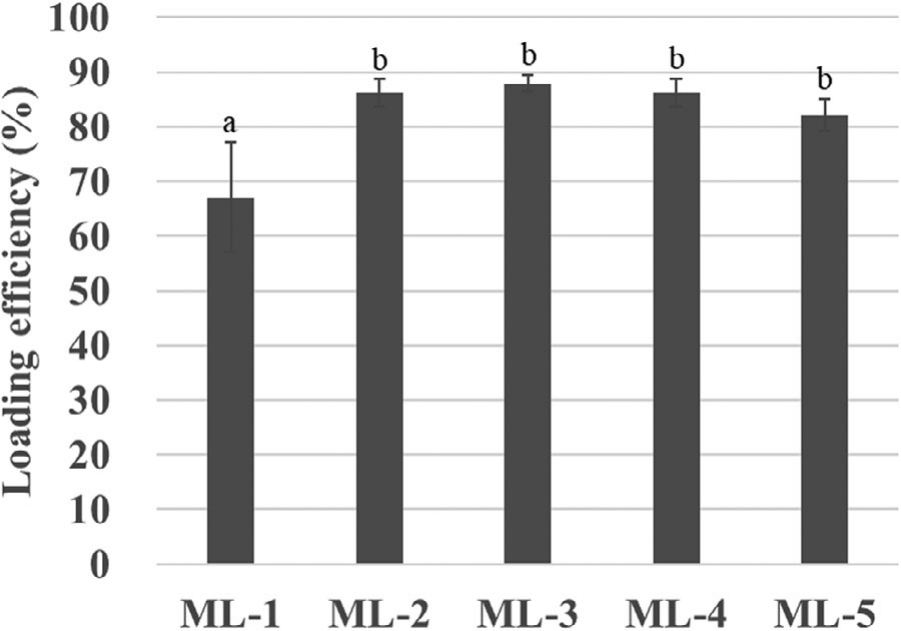
Encapsulation efficiencies of MLs containing retinol; different letters (a and b) indicate significant differences (*p* < 0.05).

The retinol encapsulation efficiency of liposome formulations according to the HL:MEL mass ratio is shown in Figure 2. The encapsulation efficiencies of ML‐1, ML‐2, ML‐3, ML‐4, and ML‐5 were measured as 67.1 ± 10.1%, 86.3 ± 2.5%, 87.9 ± 1.4%, 86.3 ± 2.3%, and 82.1 ± 2.9%, respectively.

**FIGURE 3:**
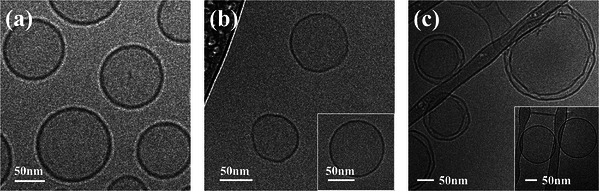
Cryo‐TEM images of (a) ML‐1, (b) ML‐4, and (c) ML‐5 (The scale bars represent 50 nm).

The encapsulation efficiency of the active ingredients in liposomes is affected by the surfactant used [24]. The normal liposome ML‐1, which was manufactured solely with HL, exhibited the lowest encapsulation efficiency, whereas the deformable liposomes (ML‐2, ML‐3, and ML‐4) manufactured with HL and MEL exhibited encapsulation efficiencies of >85%. When MEL and HL are present in a mixed ratio, the solubility of retinol is higher than that of HL alone, suggesting that the content of retinol in the deformable liposome has increased. In particular, the hydrophobic fatty acid chains of MEL are predominantly composed of hexanoic acid (49 mol%), dodecanoic acid (23 mol%), tetradecanoic acid (19 mol%), and the unsaturated fatty acid tetradecenoic acid (9 mol%) [23]. Thus, the chain length of MEL is far shorter than the average fatty acid chain length of HL used in liposome preparation. Hence, MEL can increase the encapsulation efficiency of lipid components such as retinol by providing a flexible space in the rigid lipid bilayer membrane of liposomes composed solely of HL. Therefore, the encapsulation efficiencies of ML‐3 and ML‐4, which were composed of HL, were 87.9% and 86.3% respectively, indicating that ML had an approximately 20% higher encapsulation efficiency than the normal liposome. Although ML‐5 also exhibited a higher encapsulation efficiency (82.1%) than ML‐1, this formulation is considered to have little practical utility in encapsulation efficiency owing to the instability of liposome particles over time, as shown in Figures 1 and 3.

As mentioned previously, the CMC of MEL is approximately 10 mg/L (1.5 × 10^−5^ M), which is lower than those of other synthetic surfactants such as SDS and SDBS [24]. Thus, in formulations ML‐2 to ML‐5, it is possible that some of the MEL is not present within the ML but rather forms micelles in the solution, with some retinol being trapped inside the micelles. Therefore, it is believed that these results may be reflected in the physicochemical properties of the MLs, including the encapsulation efficiency for each formulation.

### 3.4 Deformability Indices of MLs

The deformability indices were measured to evaluate the deformability of the MLs (Table 3). The deformability index was measured as 2.44 ± 1.1 for ML‐1, 3.24 ± 1.4 for ML‐2, 3.88 ± 0.8 for ML‐3, 5.76 ± 1.9 for ML‐4, and 27.38 ± 6.5 for ML‐5.

**TABLE 3:** Deformability indices of the MLs.

Formulation code	Deformability index
ML‐1	2.44 ± 1.1^a^
ML‐2	3.24 ± 1.4^b^
ML‐3	3.88 ± 0.8^b^
ML‐4	5.76 ± 1.9^b^
ML‐5	27.38 ± 6.5^b^

Different letters (a and b) indicate significant differences (*p* < 0.05).

Conventional liposomes without edge activators have limitations in penetrating the skin [25]. The edge activator makes the lipid bilayer of deformable liposomes more flexible, increasing their deformability [26]. As mentioned previously, the normal liposome ML‐1 exhibited the lowest deformability among the liposomes we tested. Furthermore, it was confirmed that as the mass ratio of MEL used as an edge activator increased to 30%, 50%, and 70%, the deformability increased. We consider that the increase in the MEL ratio transformed the liposome membrane from a rigid membrane into a flexible membrane, increasing the deformability. Among the aforementioned HL:MEL ratios, 3:7 (corresponding to ML‐4) yielded the highest deformability index. ML‐5, which was prepared solely with MEL, had the highest deformability index among the MLs tested. However, MEL fails to exhibit the strong hydrophilic properties of phospholipids during liposome formation. Additionally, while phospholipids form a stable and rigid liposome membrane owing to hydrophobic interactions of long fatty acid chains, MEL struggles to form a stable and rigid liposome membrane, because of the short fatty acid chains. Thus, although the deformability index is high, it is challenging to maintain the stability of the liposome (Figure 1), indicating that its particle size is unstable over the 4‐week period. Then, as more time passed, liposome structures were no longer observable due to aggregation (data not presented). This suggests that a certain amount of phospholipid or HL is essential for liposome preparation when MEL is used as an edge activator.

The measured physical properties indicated that the ML‐4 formulation, with an HL:MEL mass ratio of 3:7, had a uniform particle size, a high absolute zeta potential, stability during the storage period, a high encapsulation efficiency of retinol, and a high deformability index.

### 3.5 Morphological Analysis of ML

Figure 3 shows the structure of the MLs as observed using cryo‐TEM. Figure 3a presents a cryo‐TEM image of ML‐1, showing the dense bilayer and uniform spherical shape of phospholipid liposomes. ML‐4 also exhibited a typical liposome bilayer and a spherical membrane structure with a thinner bilayer than ML‐1 (Figure 3b). ML‐5, which was made entirely of MEL, had both a liposome bilayer and multilayer structures, as well as particles of varying sizes and unstable, aggregated forms, in contrast to ML‐1 and ML‐4 (Figure 3c).

Furthermore, the thickness of the liposome bilayer observed in cryo‐TEM was approximately 4–5 nm for ML‐1, 3.5–4.5 nm for ML‐4, and 2.5–3.5 nm for ML‐5. These differences in bilayer thicknesses are likely due to the structural differences between the hydrophilic and hydrophobic groups of lecithin and MEL. MEL has significantly shorter alkyl chain and nonionic hydrophilic segment and lower hydrophilicity than HL. Therefore, as the ratio of MEL in the surfactant forming the liposome bilayer increases, the thickness of the bilayer decreases. Accordingly, the liposome membrane was thinner in ML‐4 than in ML‐1, as indicated by the cryo‐TEM images.

### 3.6 In Vitro Franz Diffusion Cell Skin Permeation of MLs

**FIGURE 4:**
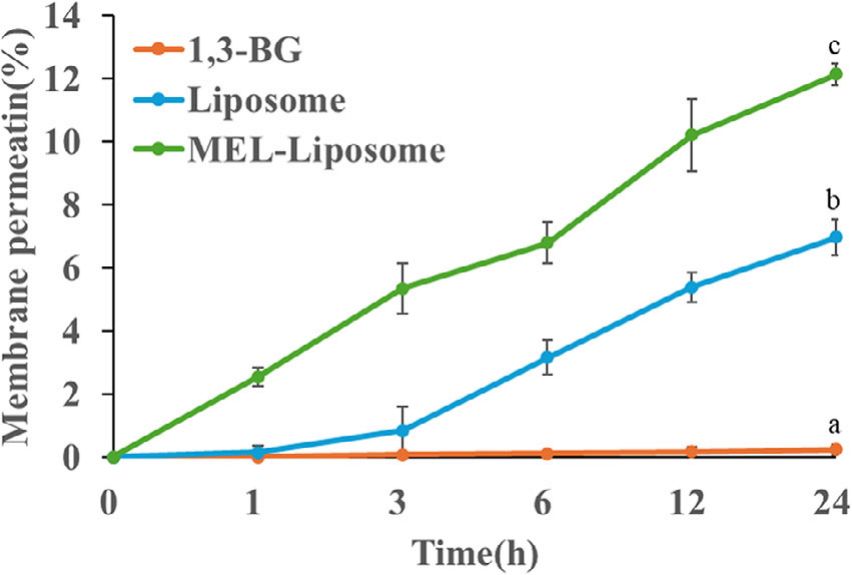
In vitro skin permeation profiles of the 1,3‐BG solution, ML‐1, and ML‐4 containing retinol over 24 h; different letters (a, b and c) indicate significant differences (*p* < 0.05).

**FIGURE 5:**
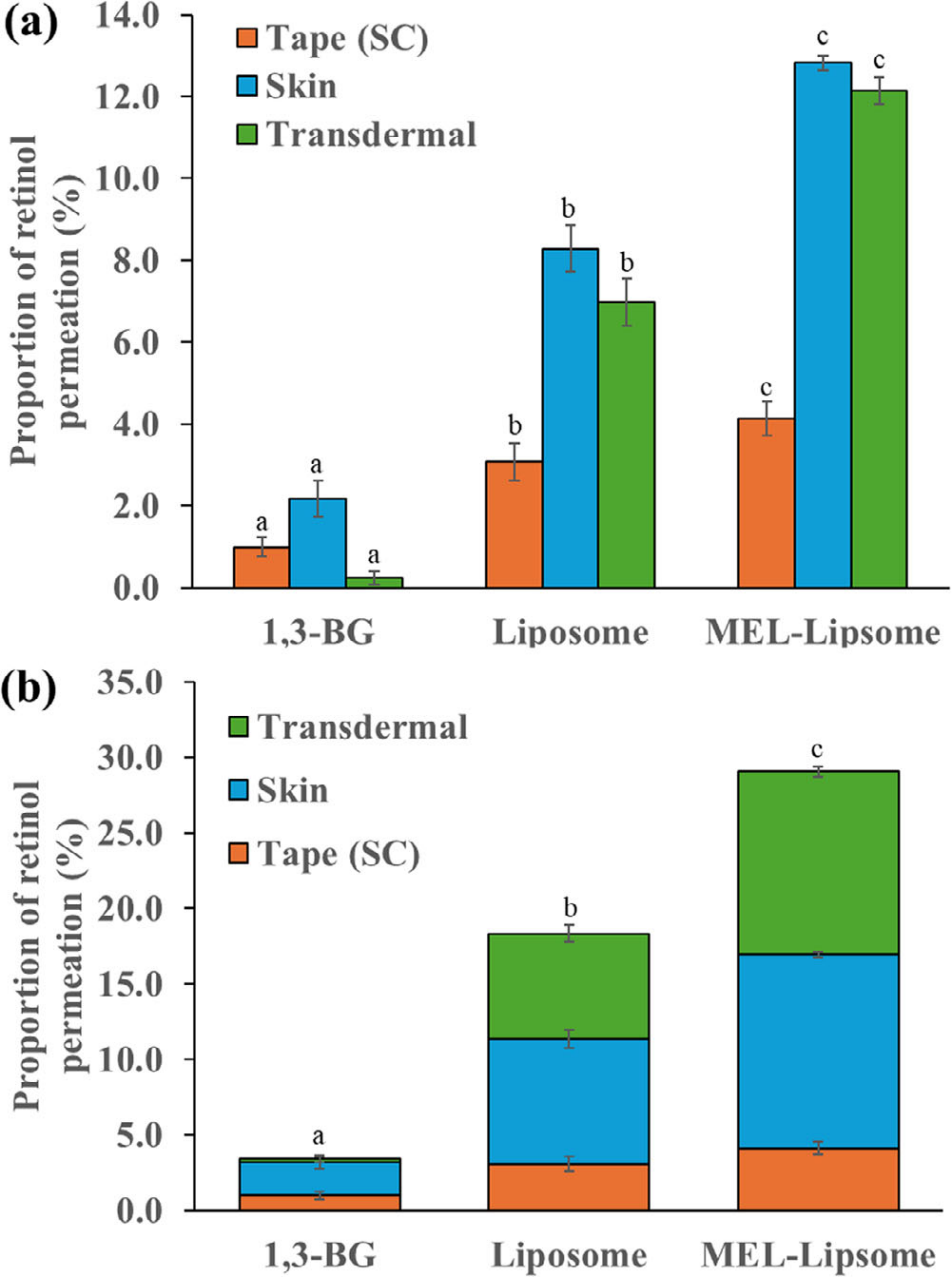
Proportions of the permeated amount of the 1,3‐BG solution, ML‐1 and ML‐4 containing retinol through human skin after 24 h of incubation (Tape: stratum corneum, Skin: epidermis and dermis, Transdermal: permeated through skin); different letters (a, b and c) indicate significant differences (*p* < 0.05).

In this study, a deformable liposome TDDS using MEL as an edge activator was developed to enhance the skin absorption of retinol, which is known as an anti‐wrinkle agent in functional cosmetics. Additionally, to investigate how the developed delivery system affects the skin absorption rate compared with the existing delivery system, the in vitro skin permeation ability was evaluated using a Franz diffusion cell (Figures 4 and 5). In the experiment, ML‐4 (HL:MEL = 3:7), which was selected as the optimal formulation, ML‐1 consisting only of HL, and a retinol solution dissolved in 1,3‐BG as a control group were used.

Figure 4 presents the measured amounts of retinol in the 1,3‐BG, ML‐1, and ML‐4 formulations that passed through the skin (transdermal) at different time points (0, 1, 3, 6, 12, 24 h). As shown, ML‐4 had the highest skin permeation rate of retinol at 24 h (12.1%), followed by ML‐1 (6.9%) and 1,3‐BG (0.2%) at 24 h.

Figure 5a shows the amount of retinol present in the stratum corneum (Tape), the amount present in the epidermis and dermis excluding the stratum corneum (Skin), and the amount that penetrated the skin (Transdermal) (Figure 5). After 24 h of skin application, the amount of retinol absorbed and present in the stratum corneum (Tape) was 1.0 %, 3.1 %, and 4.1 % for the 1,3‐BG, ML‐1, and ML‐4 formulations, respectively. Thus, the amount absorbed into the stratum corneum was the largest for ML‐4. The amount of retinol that permeated into the epidermis and dermis (Skin) was also the largest for ML‐4; it was 2.2, 8.3, and 12.8 % for 1,3‐BG, ML‐1, and ML‐4, respectively. Finally, the amount of retinol that penetrated the skin (Transdermal) was 0.2, 7.0, and 12.1 % for 1,3‐BG, ML‐1, and ML‐4, respectively. Thus, the ML‐4 formulation showed the largest amount of retinol that completely penetrated the skin (Transdermal). In the case of ML‐4 deformable liposomes, the amount of retinol absorbed into the skin layer was similar to the amount of transdermal retinol.

Figure 5b shows the combined amount of retinol in the stratum corneum (Tape), skin layer (Skin), and transdermal (Transdermal), as represented in Figure 5a. The total skin penetration of retinol for the 1,3‐BG, ML‐1, and ML‐4 formulations was 3.4%, 18.4%, and 29.0%, respectively. Among these, the ML‐4 deformable liposome showed the highest skin penetration due to its small particle size, high encapsulation efficiency and flexible liposome membrane. The addition of MEL between the lipid bilayer not only increased the lipid bilayer flexibility but also introduced MEL‐derived fatty acids, which are shorter than the phospholipid fatty acid chains. This structural modification facilitated liposome fusion with biological membranes and enhanced drug penetration efficiency owing to their ability to efficiently encapsulate drugs such as retinol [27].

Compared with the normal liposome (ML‐1) using only lecithin without MEL, ML‐4, which used MEL as an edge activator and had an HL:MEL mass ratio of 3:7, exhibited 4.5 % greater skin permeation in the skin layer (Skin = Epidermis + Dermis) and 5.1 % greater skin transit of retinol (Transdermal) (Figure 5).

In this experiment, the retinol used as a functional material exhibited anti‐aging activity such as wrinkle improvement by stimulating collagen synthesis in the dermis, inhibiting MMP expression, and reducing oxidative stress, as mentioned previously. Additionally, its derivative RA has been reported to inhibit excessive pigmentation by downregulating the expression of tyrosinase—a key enzyme in melanin synthesis—in melanocytes located in the basal layer of the epidermis and thereby inhibiting melanin production and the transfer of melanosomes to keratinocytes. Therefore, this MEL‐deformable liposome formulation (ML‐4) allowed 24.9% of retinol to pass through the skin (Skin + Transdermal), efficiently delivering retinol to the skin layer at a content that was 9.6% greater than the relative penetration amount of 15.3% for the conventional liposome (ML‐1). In this experiment, the skin penetration of retinol in the ML‐4 formulation was 29.0%. This amount is 14.5 mg/100 mL for the initial concentration of 50 mg/mL, which is a sufficient concentration to actually show efficacy in the skin. However, since improving the skin penetration of retinol is also important in cosmetics, it is thought that further research is needed.

It is believed that using this MEL‐deformable liposome as a TDDS can induce anti‐aging effects such as wrinkle improvement as well as whitening effects that inhibit hyperpigmentation.

## 4 Conclusions

In this study, to enhance the skin absorption of retinol, a functional cosmetic ingredient known for its wrinkle‐improving properties, deformable liposome formulations loaded with retinol were prepared using HL and MEL as an edge activator at weight ratios of HL:MEL = 10:0, 7:3, 5:5, 3:7, and 0:10. Among the five formulations (ML‐1 to ML‐5), ML‐4, with a weight ratio of HL:MEL = 3:7, was identified as the optimal deformable liposome formulation based on these characteristics. The morphological structure of the liposome was examined using cryo‐TEM, comparing ML‐4 with the conventional liposome ML‐1 (without MEL) as a control. The skin penetration ability of retinol in each formulation was evaluated. Cryo‐TEM observations confirmed that ML‐4 maintained a stable liposome membrane with a distinct lipid bilayer, similar to ML‐1. The retinol skin penetration ability of the ML‐4 deformable liposome formulation was more than 10% higher than that of the conventional liposome (ML‐1). These results indicate that the retinol loaded ML‐4 deformable liposome formulation significantly enhances the skin penetration and absorption of functional cosmetic ingredients.

In the deformable liposome studied previously, synthetic surfactants such as polyethylene glycol (PEG) nonionic surfactants were used as edge activators and were manufactured. PEG‐based synthetic surfactants are rarely used in the cosmetics industry at present due to safety issues. Therefore, in this study, we prepared deformable liposome using MEL bioactive surfactant, which is excellent in physicochemical properties and efficacy as well as safety and compatibility with cosmetics applications, and developed a new formulation that allows retinol, an anti‐aging agent, to penetrate deep into the skin, which is considered to be very significant.

Therefore, the newly developed MEL‐deformable liposome formulation shows great potential for sustainable applications in cosmetics and development of a TDDS platform for functional skin care.

## Ethics Statement

This study was conducted in accordance with the ethical guidelines of the Society of Cosmetic Scientists of Korea (SCSK). Human cadaver skin used in this research was obtained through Seedgroup Derma:Lab^TM^ (Seoul, Republic of Korea), a certified tissue procurement organization. The supplier provided documentation confirming that the tissue was legally donated by a deceased individual with appropriate body donation authorization. Written informed consent for tissue donation was obtained from the donor's next of kin and documented in the “Authorization for Tissue Donation” form, which permits the use of the donated tissue for research and educational purposes. The samples were provided in a fully de‐identified manner, and no donor‐identifiable information was accessible to the authors. According to the U.S. Common Rule (45 CFR 46), a human subject is defined as a living individual; therefore, research involving cadaveric tissue does not constitute human subjects research and does not require Institutional Review Board (IRB) approval.

## Conflicts of Interest

The authors declare that they have no known competing financial interests or personal relationships that could have appeared to influence the work reported in this paper.

## Data Availability

The data that support the findings of this study are available from the corresponding author upon reasonable request.
